# Good vaccination practice: it all starts with a good vaccine storage temperature

**DOI:** 10.1186/s40813-017-0071-4

**Published:** 2017-12-04

**Authors:** Frédéric Vangroenweghe

**Affiliations:** Elanco Animal Health, BU Swine & Poultry, Plantijn en Moretuslei 1, 2018 Antwerpen, Belgium

**Keywords:** Good vaccination practice, Cold chain, Vaccine storage temperature, On-farm refrigerator

## Abstract

**Background:**

Recent introduction of strategies to reduce antibiotic use in food animal production implies an increased use of vaccines in order to prevent the economic impact of several important diseases in swine. Good Vaccination Practice (GVP) is an overall approach on the swine farm aiming to obtain maximal efficacy of vaccination through good storage, preparation and finally correct application to the target animals. In order to have a better insight into GVP on swine farms and the vaccine storage conditions, a survey on vaccination practices was performed on a farmers’ fair and temperatures in the vaccine storage refrigerators were measured during farm visits over a period of 1 year.

**Results:**

The survey revealed that knowledge on GVP, such as vaccine storage and handling, needle management and injection location could be improved. Less than 10% had a thermometer in their vaccine storage refrigerator on the moment of the visit. Temperature measurement revealed that only 71% of the measured refrigerators were in line with the recommended temperature range of +2 °C to +8 °C. Both below +2 °C and above +8 °C temperatures were registered during all seasons of the year. Compliance was lower during summer with an average temperature of 9.2 °C while only 43% of the measured temperatures were within the recommended range.

**Conclusions:**

The present study clearly showed the need for continuous education on GVP for swine veterinarians, swine farmers and their farm personnel in general and vaccine storage management in particular. In veterinary medicine, the correct storage of vaccines is crucial since both too low and too high temperatures can provoke damage to specific vaccine types. Adjuvanted killed or subunit vaccines can be damaged (e.g. structure of aluminiumhydroxide in adjuvans) by too low temperatures (below 0 °C), whereas lyophilized live vaccines are susceptible (e.g. loss of vaccine potency) to heat damage by temperatures above +8 °C. In conclusion, knowledge and awareness of GVP and vaccine storage conditions are crucial under practical field conditions in swine herds. Focus on a correct on-farm vaccine storage is part of the responsible veterinarians’ guidance in order to obtain the required vaccine efficacy.

## Background

Since 2007, stringent measures to reduce antibiotic consumption by 50% in food producing farm animals, including pigs, were imposed in The Netherlands [1]. In Belgium, the Knowledge Center for Antimicrobial Consumption and Resistance in Animals (AMCRA) has formulated ambitious targets for the reduction of antibiotic use in farm animals by 2020. Due to this antibiotic reduction, a major increase in the use of vaccinations against most currently present swine pathogens, such as *M. hyopneumoniae* (*M.hyo*), Porcine Reproductive and Respiratory Syndrome virus (PRRSv), Porince Circovirus type 2 (PCV-2), *Actinobacillus pleuropneumoniae* (App) and *Haemophilus parasuis* (Hps) has been observed. In the past, vaccinations have contributed to decreasing serious outbreaks by preventing incidence and propagation of contagious diseases in advance [2]. Vaccination programs are cost-effective in preventing outbreaks and spread of vaccine-preventable diseases [3].

To obtain maximal results from the applied vaccination strategies and to ensure optimal potency of vaccines used in veterinary medicine [4], the vaccines have to be handled with care from production through distribution and on-farm storage until application to the target animals under practical field conditions. The World Health Organisation (WHO) recommends that all vaccines should be stored at between +2 °C and +8 °C at all segments of the cold chain [4]. The need to address this challenge has become increasingly important due to the introduction of new and more expensive combined vaccines that are at risk of damage from heat and/or freeze exposure [5–8].

Maintenance of the cold chain during transport and storage by the end user has been shown to be critically important [4, 9]. In human medicine, several studies were conducted towards general awareness of the importance of cold chain management and the risks of vaccine storage outside the current WHO recommendations of between +2 °C to +8 °C [10]. Depending on the type of vaccine, storage both under too cold (below 0 °C) or too hot (above +8 °C) temperatures can be detrimental for the vaccine potency and its subsequent immunological characteristics following administration to the patient [11]. For adjuvanted vaccines, such as killed (e.g. *M.hyo*, PCV-2, Hps) and subunit (App) vaccines, which are also frequently used in veterinary medicine, storage under 0 °C may cause an irreversible damage to the structure of the adjuvant, resulting in a decreased immunogenicitiy of the vaccine [11–14]. The shake test is the only test with 100% sensitivity, 100% specificity and 100% predictive value to determine whether aluminium-adjuvanted freeze-sensitive vaccines have been affected by freezing [15]. Live vaccines (e.g. PRRSv, *E. coli*), on the other hand, are more prone to damage due to exposure to temperatures above +8 °C, resulting in loss of vaccine potency [11]. Programs focusing on education and improved awareness of the different aspects of vaccine handling and storage by the end users have shown a significant improvement of overall vaccine storage quality [16–18].

Good Vaccination Practice (GVP) is a terminology summarizing the entire procedure from on-farm vaccine receipt until the administration of the vaccine to the target animals, comprising vaccine storage, vaccine preparation for administration and the vaccine administration equipment (including needles, syringes, needleless devices, …) [19]. Essential elements to check for within the GVP are the refrigerator itself (type, maximal age [20], stable power supply [19]), including accurate knowledge on basic storage principles (first-in first-out (FIFO) principle [19], no vaccine in the door shelves [19], correct range of storage temperature [19], no freezing [11–14]), followed by planning of the vaccination session and vaccine preparation before administration (including the acclimatization of the vaccine to room temperature (+18-20 °C) before administration). For the administration itself several aspects should be taken into account such as needle type (length and diameter adapted to the target animal group) [19] and exact injection location. Subsequent management and conservation of bottles that have been opened but not entirely used is also an important issue.

The aims of the present study were first to obtain data on the current knowledge of swine farmers of the most important principles of GVP; and second to measure on-farm vaccine storage temperature at the level of the vaccine refrigerator in order to monitor the current vaccine storage situation on swine farms in Belgium and The Netherlands.

## Methods

### Survey on level of knowledge concerning good vaccination practices

In order to quantify the level of knowledge concerning the key essentials of GVP, a survey of 8 questions on different aspects of GVP was organized on a 3-day farmers’ fair in 2015 (LIV Hardenberg, Hardenberg, The Netherlands) (Table 1). The multiple choice questions (Table 2) were presented to 50 sow farmers in The Netherlands with at least 200 sows that were willing to cooperate in the questionnaire. The average respondent had 568 (±80) sows and half of them had additional farm personnel assisting the vaccination process.

**Table 1:** Survey questionnaire on GVP at a farmers’ fair in The Netherlands

N°	Question
1	Are vaccination tasks performed by the swine farmer himself or with the help of other farm personnel?
2	What is the temperature range for storage of vaccines in the refrigerator?
3	What time interval is needed to warm a vaccine from storage temperature to room temperature (18-20 °C)?
4	When freezing of vaccines occurs during storage, what is the consequence?
5	How long can a vaccine bottle after first use still be stored without quality decrease and with full vaccine efficacy?
6	What is the optimal needle management?
7	What is the ideal dimension (length & diameter) for vaccination of piglets during the first week of life?
8	What is the correct injection site for vaccines in the neck region?

**Table 2:** Questionnaire responses (*n* = 50) on GVP knowledge

N°	Question	Response (%)
1	Are vaccination tasks performed by the swine farmer himself or with the help of other farm personnel?	
	a. Yes	50%
	b. No	50%
2	What is the temperature range for storage of vaccines in the refrigerator?	
	a. 0–5 °C	14%
	**b. 2–8 °C**	**80%**
	c. Doesn’t matter as long as refrigerated	6%
3	What time interval is needed to warm a vaccine from storage temperature to room temperature (18-20 °C)?	
	a. 1 h	76%
	**b. 5 h**	**18%**
	c. 10 h	2%
	d. The day before vaccination	4%
4	When freezing of vaccines occurs during storage, what is the consequence?	
	**a. Antigen in the vaccine damaged**	**78%**
	b. No negative effect on immunity	16%
	c. Stronger immune response	6%
5	How long can a vaccine bottle after first use still be stored without quality decrease and with full vaccine efficacy?	
	**a. 24 h**	**62%**
	b. 1 week	28%
	c. 1 month	4%
	d. Until expiry date	6%
6	What is the optimal needle management?	
	a. Needles until broken	6%
	b. Disposable needle every 10 litters	36%
	**c. Disposable needle every litter**	**58%**
7	What is the ideal dimension (length & diameter) for vaccination of piglets during the first week of life?	
	**a. Length 9 mm, diameter 0.8 mm**	**58%**
	b. Length 12 mm, diameter 1.0 mm	22%
	c. Length 16 mm, diameter 0.8 mm	20%
8	What is the correct injection site for vaccines in the neck region?	
	a. In the lower region of the neck	18%
	**b. 2 fingers behind the ear**	**52%**
	c. Just in front of schoulder	30%

Correct answers are in bold

### On-farm measurement of vaccine storage refrigerator temperature

The actual refrigerator temperature was measured in 126 swine farm vaccine storage refrigerators during a consultative farm visit in Belgium and The Netherlands. The number of farms per season is given in Table 3. Every refrigerator was only measured once, since multiple measurement over time would bias the study data through the increased awareness following the first measurement. The digital thermometer sensor (MOXX Thermometer; TFA® Dostmann GmbH & Co., Wertheim, Germany) was installed in the refrigerator in a standardized way:

1. The sensor was inserted into a cardboard packaging box of a veterinary medicinal product (VMP) present in the refrigerator
2. The VMP package was positioned in the central part of the body of the refrigerator (not the door shelf, not the upper nor lower shelf)
3. Temperature measurement was allowed for at least 45 min
4. The actual refrigerator temperature was noted including date, season and country of measurement.

**Table 3:** Number of measured farms, average (±SEM) vaccine storage temperature and distribution (%) of on-farm measured refrigerator temperatures in specific temperature category: below +2 °C, between +2 °C and +8 °C, above +8 °C

	# farms per season	Average (± SEM) t° per season	Below +2 °C	Between +2 °C and +8 °C	Above +8 °C
S1, winter	26	6.5 ± 0.64	4	77	19
S2, spring	43	6.4 ± 0.33	0	81	19
S3, summer	23	9.2 ± 1.11	4	43	52
S4, autumn	34	6.5 ± 0.47	6	71	24
*Summary*	*126*		*3*	*71*	*26*

Additionally, it was registered if a thermometer or other temperature monitoring system was already present in the vaccine storage refrigerators included in the study.

### Statistical analysis

Results from the survey were reported as descriptive data with the % of respondents per answer category.

Measured temperatures were categorized based on the season they were measured: S1 (winter; 21/12 – 20/3), S2 (spring; 21/3 – 20/6), S3 (summer; 21/6 – 20/9) and S4 (autumn; 21/9 – 20/12). Since the vaccine storage temperature data were single point measurement of different farms in different seasons, data were reported as descriptive data, including average (± SEM) over season. The distribution of the vaccine storage refrigerator temperatures over the year was plotted in a histogram with intervals of 3 °C.

## Results

### Survey on level of knowledge concerning good vaccination practices

In total, 50 valid survey responses were collected during the 3-day farmer event. The summary of the responses is shown in Table 2.

The most important observation concerning vaccine storage were that only 80% of the respondents could identify the temperature range of +2 °C to +8 °C as the recommended temperature range for on-farm vaccine storage. There were also 22% of the respondents that did not realize freezing had a significant impact on the subsequent vaccine efficacy. Other questions related to GVP revealed that needle management in general and needle length per animal category and site of injection in particular were not always quite clear to swine farmers. The interval needed to get a vaccine at room temperature (+18 °C) ready for injection was also not very clear.

### On-farm measurement of vaccine storage refrigerator temperature

Only 12 (9.5%) vaccine storage refrigerators already had a thermometer present at the moment of the farm visit.

**Fig. 1:**
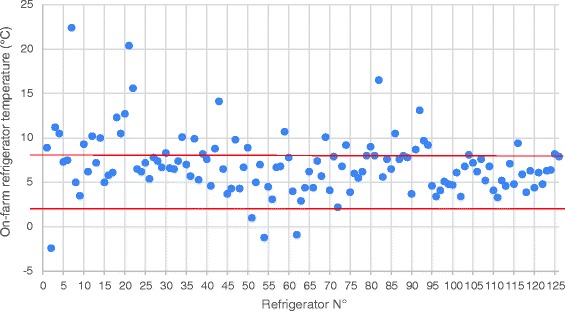
On-farm refrigerator temperatures (individual data points) measured during 2016-2017 in Belgium and The Netherlands. Red lines indicate lower (+2 °C) and upper (+8 °C) limits of recommended temperature

**Fig. 2:**
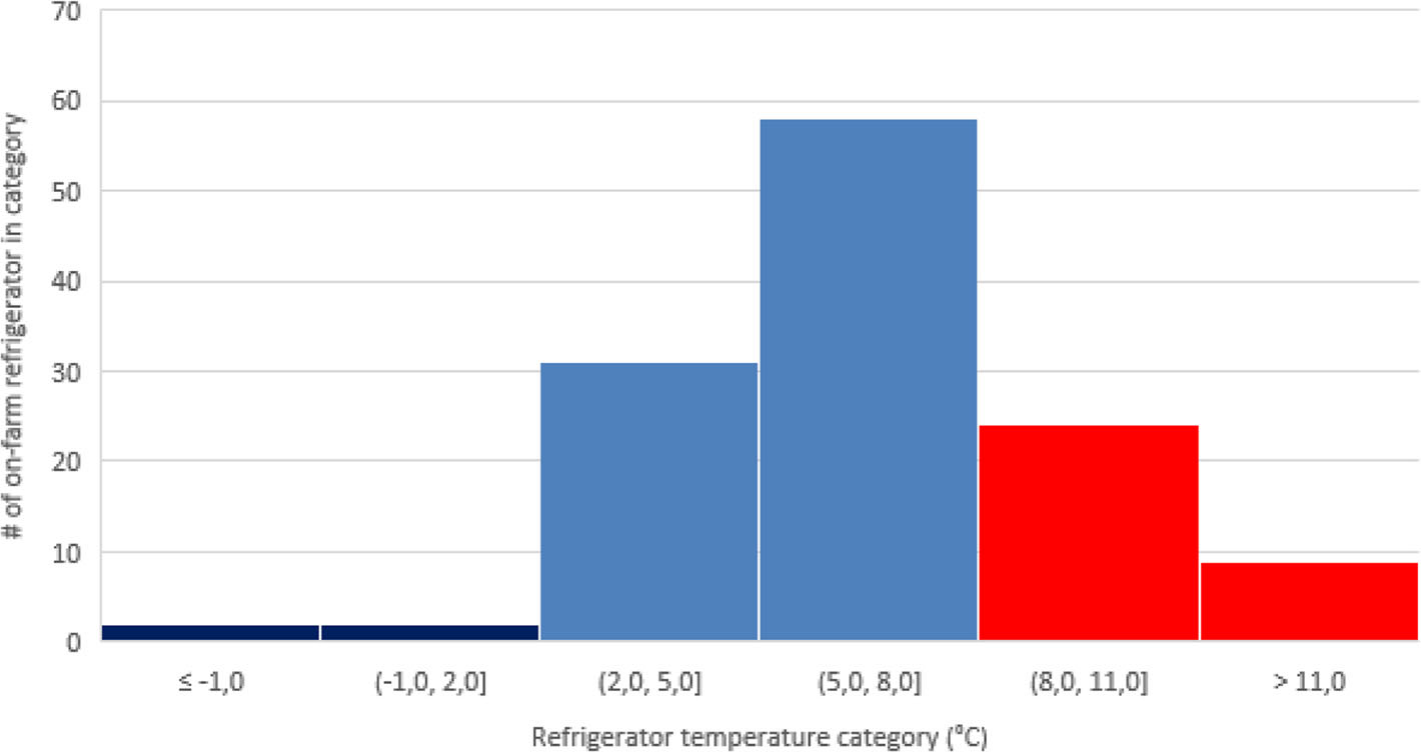
Distribution of 126 refrigerator temperatures collected on-farm in Belgium and The Netherlands during 2016-2017

The variation in vaccine storage temperature among the 126 on-farm measurement is shown in Fig. 1. It is apparent that only in 4 cases, the vaccine storage temperature was below the +2 °C, with sub-zero temperature in 3 cases. In total, 33 cases exceeded the upper range of +8 °C. Most of these events (73%) concerned slight breaches between +8 °C and +11 °C (Fig. 2).

Analysis of seasonal patterns revealed significant differences among season with a statistically higher temperature (9.2 ± 1,1 °C) during the summer (S3) as compared to other seasons. During summer (S3), 52% of the on-farm vaccine storage refrigerators exceeded the upper limit of +8 °C, whereas in other seasons, this percentage varied between 19% (winter; S1 and spring; S2) and 24% (autumn; S4) (Table 3). In total, only 71% of the measured temperatures were within the WHO-recommended range.

## Discussion

Even in human medicine, compliance to the WHO recommendations on vaccine storage is a difficult issue [20]. In our survey, 80% of the participants could state the recommended temperature range of between +2 °C and +8 °C. This is much higher than in another study conducted in human medicine, which obtained a score of only 16% [21]. We have to realize that our measurements were performed at a lower level in the cold chain as compared to most studies performed in a human environment, where vaccine storage refrigerators at the physicians’ office were monitored. Nevertheless, we have to emphasize that on-farm vaccine storage is a shared responsibility of both the swine farmer and its veterinarian responsible for on-farm health management and epidemiological surveillance. Two other point of interest in vaccine storage could also be improved this way. First, the fact that frozen vaccines lose their immunological activity and secondly, opened bottles that are not entirely used should not be kept too long under cooled storage after its first use. In this aspect, indication of the date of first use on the bottle would mean a positive evolution.

Another interesting result from our survey is the fact that 76% of the respondents apparently inject the vaccines at a too cold temperature, which might cause injection problems, especially in the case of oil-based vaccines. Current knowledge on the needle specifications for the target animal group to be vaccinated were also quite low (58%) as well as needle management and general hygiene measures to omit transmission of pathogens from one litter to another through injections. It has indeed been shown that e.g. PRRSV can be transmitted among pigs in a swine herd through injection needles [22]. In human medicine, the aspects of needle management and hygiene are quite well defined and carefully followed [19], since stringent protocols are in place to assure maximal prevention of disease between patients [19].

Cold chain monitoring can be performed using different temperature measurement tools, such as cold chain measuring cards [10], digital thermometers [11] including minimum-maximum thermometers and electronic temperature loggers [11]. However, a thermometer only provides a snapshot of the temperature at the point in time when it is checked and can therefore not be considered a long-term appropriate monitoring tool [23], unless outer limits (minimum/maximum) are registered as in the case of a minimum-maximum thermometer. When the temperature is checked and the value is found to be in the range of +2 °C to +8 °C, farmers and vets may erroneously conclude that the vaccine storage on-farm is safe, however, this snapshot measurement is not covering any deviations observed during the rest of the daytime/nighttime period. Therefore, in the present study, we used a minimum-maximum thermometer, in order to provide a tool to the farmer and the veterinarian to emphasize their continuous awareness on the importance of keeping the on-farm vaccine refrigerator within the recommended range (+2 °C till +8 °C) for vaccine storage.

Practices exposing vaccines to both high (> +8 °C) and sub-zero temperatures (< 0 °C) are widespread in both developed and developing countries at all levels of the human health system [8, 21, 24–26]. A recent review on vaccine freezing highlights that accidental freezing is widespread and occurs across all segments of the cold chain [20]. In human medicine, between 14 and 35% of refrigerators or transport shipments were found to have exposed vaccines to temperatures below zero. From our study, it is clear that, although the temperature measurement was a snapshot measurement at the time of the farm visit, only 4% of the refrigerators showed a temperature below the acceptable lower limit of +2 °C at the moment of our visit.

In human medicine, it has been shown that compliance and follow-up of the correct refrigerator temperature was higher when awareness of all stakeholders was kept up-to-date [16, 17, 27]. For example, the knowledge on the fact that heat is harmful to vaccines was rather high (75–100%) [27], whereas the awareness by swine farmers that freezing was also harmful to some vaccines was very low (22.7–44.4%) [27]. From our survey, it is also clear that knowledge and awareness on vaccine storage practices in the broad sense are inadequate. Another point of attention to increase the awareness of continuous monitoring of vaccine storage refrigerator temperature is the daily recording of measured temperature [3, 18].

In our study, 71% of the measured vaccine storage refrigerators were in the recommended temperature range. This is in accordance with other studies in human medicine which showed between 68.1% [27] and 83% [3] of the vaccine refrigerators within the recommended range. The observation that the older the refrigerator, the higher the mean temperature [27], however, bears us some concerns from a veterinary point of view. Personal observations within our area reveal that many refrigerators used for on-farm vaccine storage have been ‘recycled’ from previous service in home or office kitchens. Therefore, their average age might be well above 12 years, which has been shown to be more likely to result in inappropriate temperatures (52.2% risk) [27]. Unfortunately, we were unable to register the exact age of on-farm vaccine refrigerators in our study, due to lack of reliable data on the farm.

In our study, there were few (< 10%) on-farm vaccine refrigerators which had a thermometer present at the moment of the farm visit. This is somehow in accordance with another study in human medicine where the presence of a thermometer was 11% [27]. However, a more recent study in physicians practices in the same area obtained much higher compliance with an 83% presence of a temperature monitoring device [3].

Another potential solution for improved vaccine efficacy under difficult storage conditions could be the development of more thermostable vaccines by the pharmaceutical industry [28, 29]. However, this possibility is not yet available, and therefore, until then, compliance with the recommended vaccine storage temperatures throughout the entire cold chain remains key to maximize the efficacy of all vaccines used to prevent infectious diseases.

Programs designed to supervise and improve all aspects of vaccine storage management among physicians-professionals have demonstrated significant improvement [16, 17, 27]. Moreover, the appointment of a local responsible person for the vaccine storage resulted in higher odds that refrigerator temperature was kept within the recommended range and the refrigerator was used for vaccine storage only [30].

Taking into consideration the need of compliance with WHO recommendations on vaccine storage, some practical guidelines on vaccine storage on farm level are as following:

- ✓ No vaccine storage in the door compartment to omit the larger temperature variations that occur each time the door is opened [3].
- ✓ No use of refrigerators equipped with an upper freezing compartment.
- ✓ No storage of other materials (food, drinks, …) in vaccine storage refrigerators to reduce the number of times the refrigerator is opened during daytime [3].
- ✓ Use bottles filled with water to reduce the temperature variation within the refrigerator when the volume is not totally filled with vaccines [31].
- ✓ Avoid older refrigerators (> 12 years old) since they have a much higher risk (52.5%) of inappropriate temperatures [27].
- ✓ Position the thermometer in the central part of the refrigerator to continuously monitor the vaccine storage temperature to improve awareness of its importance [16, 17, 27].
- ✓ Perform daily control and monitoring of vaccine storage temperature at the same timepoint [3, 18].

## Conclusions

In conclusion, on-farm vaccine storage at swine farms in Belgium and The Netherlands complied in only 71% of the cases with the recommended range between +2 °C and +8 °C. The general knowledge and awareness on issues concerning vaccine storage and GVP in a broader context show room for improvement through continuous sensibilisation and practical on-farm training of farmers and their farm personnel by the responsible farm veterinarian or other external consultants.

## Abbreviations

AMCRA: Knowledge Center for Antimicrobial Consumption and Resistance in Animals
ANOVA: Analysis of variance
App: *Actinobacillus pleuropneumoniae*
FIFO: first-in first-out
GVP: Good Vaccination Practice
Hps: *Haemophilus parasuis*
*M.hyo*: *Mycoplasma hyopneumoniae*
PCV-2: Porcine Circo Virus type 2
PRRSv: Porcine Reproductive and Respiratory Syndrome virus
VMP: Veterinary medicinal product
WHO: World Health Organisation
